# Maternal Personality and Child Temperamental Reactivity: Differential Susceptibility for Child Externalizing Behavioral Problems in China

**DOI:** 10.3389/fpsyg.2018.01952

**Published:** 2018-10-12

**Authors:** Shufen Xing, Xin Gao, Xia Liu, Yuanyuan Ma, Zhengyan Wang

**Affiliations:** ^1^Department of Psychology, Capital Normal University, Beijing, China; ^2^Institute of Developmental Psychology, Beijing Normal University, Beijing, China

**Keywords:** maternal personality, temperamental reactivity, externalizing behavioral problems, differential susceptibility, diathesis-stress model

## Abstract

It is important to identify the developmental antecedents of externalizing behavioral problems in early childhood. The current study examined the main effects of maternal personality and its interactive effects with child temperamental reactivity in predicting child externalizing behavioral problems, indicated by impulsivity and aggression. This study was composed of 70 children (*M_*age*_* = 17.6 months, *SD* = 3.73) and their mothers. The results showed that maternal agreeableness was negatively associated with child impulsivity. Child temperamental reactivity moderated the effect of maternal conscientiousness on child impulsivity in support of the differential susceptibility model. Specifically, for highly reactive children, maternal conscientiousness was negatively associated with child impulsivity whereas this association was non-significant for low reactive children. Child reactivity also moderated the contribution of maternal neuroticism to child impulsivity. That is, maternal neuroticism was negatively associated with impulsivity, only for highly reactive children.

## Introduction

### Parental Personality and Children’s Externalizing Behavioral Problems

Child externalizing behavioral problems, such as distractibility, impulsivity, and defiance, is an important topic in child development (Cormier, 2008). Epidemiological research suggests that 15–20% children exhibit social, emotional and behavioral problems (Van Hulle et al., 2007). In general, externalizing behavioral problems often have an onset in infancy (Keenan and Wakschlag, 2000) and externally problematic children tend to have difficulties at school, such as high drop-out and low attendance rates (Bulotskyshearer and Fantuzzo, 2011), behavioral disruption and delinquency (Coie and Dodge, 1998). In addition, Calkins et al. (1999) reported that high levels of externalizing behavioral problems were often precursors to developmental disorders, including attention deficit hyperactivity disorder (ADHD) and oppositional defiant disorder (ODD). Hereby, identifying the developmental antecedents of externalizing behavioral problems in early childhood is crucial in understanding children’s behavioral wellbeing.

*The ecological niche model* of development proposes three interactive subsystems: (a) the physical and social setting where children live, (b) culturally regulated customs of child care, and (c) psychology of the caretakers which directs parental strategies in childrearing and these three subsystems interact with each other in organizing the child’s developmental experience (Super and Harkness, 1986). Accordingly, parental personality, as a defining factor in the psychology of caretakers, largely determines parental expectations and behaviors and thus leads to children’s socio-emotional development. Indeed, literature has revealed significant associations between parental personality and children’s externalizing behavioral problems (Prinzie et al., 2004, 2005; Oliver et al., 2009; Koutra et al., 2017). For instance, Prinzie et al. (2004, 2005) reported that the higher maternal neuroticism, a tendency to experience negative emotions, the fewer externalizing problems exhibited in school-aged children. In addition, maternal trait anxiety and neuroticism were positively associated with four-year-old children’s behavioral difficulties (Koutra et al., 2017). To date, previous studies on the effects of maternal personality in child development have typically been conducted in school-aged children, with little attention paid to younger children.

### The Moderating Role of Children’s Temperamental Reactivity

Temperamental reactivity, as one comprehensive aspect temperamental characteristic, refers to the individual’s sensitivity to external stimulation and the intensity of his/her reaction in response (Rothbart and Bates, 2006). Temperamental reactivity is an evolutionary characteristic that underlies the reactivity of one’s neural systems. That is, highly reactive children tend to be sensitive to environmental changes and to experience strong arousal (Ramchandani et al., 2010). Several studies have revealed that child temperamental reactivity plays a moderating role in the relations between family environmental factors and child developmental outcomes (Velderman et al., 2006; Ramchandani et al., 2010; Den Berg and Bus, 2014; Xing et al., 2016).

**FIGURE 1:**
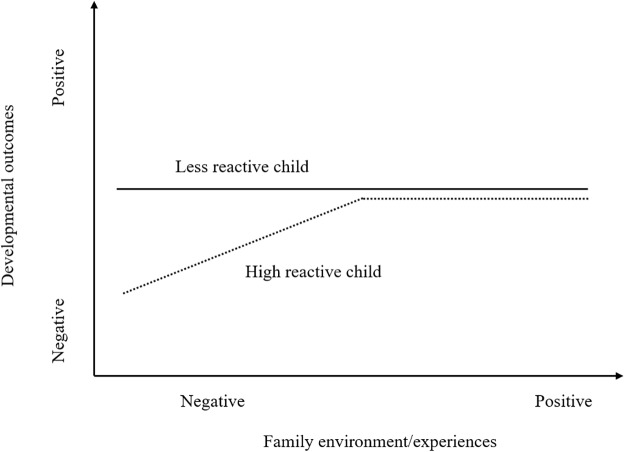
Diathesis-stress model. The highly reactive child is more susceptible to negative environment conditions. Adapted from Bakermans-Kranenburg and Ijzendoorn (2007).

**FIGURE 2:**
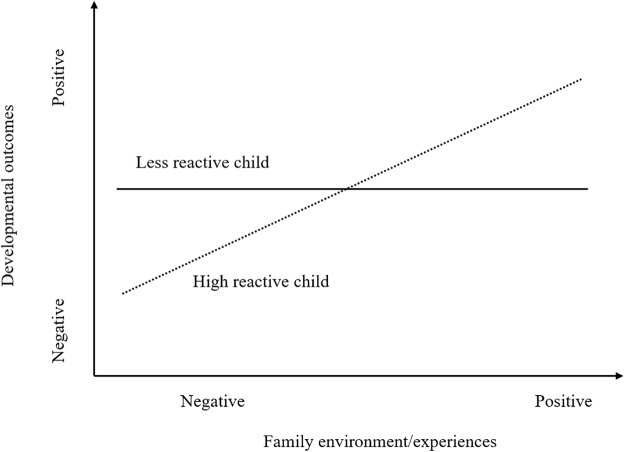
Differential susceptibility model. The highly reactive child is more susceptible to both negative and positive environment conditions. Adapted from Bakermans-Kranenburg and Ijzendoorn (2007).

In addition to the ecological niche model, two alternative models can be used to interpret the moderating role of child reactivity in the associations between family experiences and child development. *The diathesis-stress model* regards high temperamental reactivity in children as a characteristic of ‘vulnerability’, and mainly focuses on the implications of adverse environment for the development in vulnerable children. As presented in **Figure 1**, highly reactive children are especially susceptible to poor experiences at home (e.g., child maltreatment) and exhibit worse outcomes than less reactive children (Belsky and Pluess, 2009). However, the diathesis-stress model suggests no significant differences in the influence of favorable environment between highly reactive and low reactive children (Ellis et al., 2011). Relative to the diathesis-stress model, *the differential susceptibility model*, shown in **Figure 2**, further suggests that high reactivity functions as an agent of plasticity or susceptibility (Belsky, 1997). Specifically, compared to low reactive counterparts, highly reactive children are not only more vulnerable to negative environment, but also more susceptible to positive environmental effects. In other words, the reactive children are more affected by environmental factors for better or for worse (Belsky and Pluess, 2009, 2013). Based on this model, Ramchandani et al. (2010) investigated the longitudinal interactive effects between infant reactivity and paternal involvement on prosocial behaviors and behavioral problems later in childhood. The findings revealed that highly reactive girls showed significantly fewer behavioral problems and more prosocial behaviors when fathers were highly involved in childrearing and the opposite held true when fathers were least involved, supporting the differential susceptibility model. Similarly, Xing et al. (2016) found that infant reactivity moderated the effects of caregivers’ sensitivity on infants’ behavioral problems in a manner consistent with the differential susceptibility model. Taken together, literature indicates that parent-child experiences may be related with child developmental consequences in different manners, contingent on the child temperamental reactivity.

In addition to parental behaviors, recent studies also examined how the effects of parental personality on child development were moderated by child temperamental characteristics (Achtergarde et al., 2015; Cipra, 2018; Thartori et al., 2018). For instance, Thartori et al. (2018) reported that adolescents’ inhibitory control buffered the adverse effect of maternal irritability on their externalizing problems. That is, well-controlled adolescents appear to be less behaviorally problematic than others when experiencing mother’s strong irritability. In addition, Cipra (2018) found a similar role of child temperamental adaptability in moderating the effects of maternal neuroticism on children’s peer relations in kindergarten. To our best knowledge, there were no studies examining the moderating role of child reactivity in the associations between maternal personality and child externalizing behavioral problems, particularly in early childhood. The current study sought to fill the gap in the field, adopting the perspective of the differential susceptibility model.

### The Purpose of This Study

In summary, the present study mainly examined two research questions. Previous studies about maternal personality and child externalizing behavioral problems have been mostly conducted in school-aged children, so this study aimed to examine the effects of maternal personality on child externalizing behavioral problems in a sample of children aged 12 to 24 months. The Big Five is a useful framework to describe individual differences in non-clinical samples (Prinzie et al., 2004). The Big Five personality traits have been traditionally labeled as follows: (a) Extraversion. People with a high level of extraversion are talkative, assertive, and energetic, (b) Agreeableness. Agreeable people tend to be good-natured, cooperative, and trustful, (c) Conscientiousness. Conscientious individuals are orderly, responsible and dependable, (d) Neuroticism describes a tendency to be easily distressed, (e) Openness applies to people who are imaginative and independent-minded (Prinzie et al., 2005). We hypothesized that maternal Agreeableness, Extraversion and Conscientiousness would be negatively related to child externalizing behavioral problems, whereas maternal Neuroticism, and Openness would be positively related to child externalizing behavioral problems. The second purpose was to test whether the child temperamental reactivity would moderate the relations between maternal personality and child externalizing behavioral problems. According to the exiting research, children with high reactivity were more susceptible to negative or positive family factors than others because of their sensitive nervous systems (Velderman et al., 2006; Ramchandani et al., 2010; Den Berg and Bus, 2014; Xing et al., 2016). Therefore, we hypothesized that maternal personality might be associated with child externalizing behavioral problems differently, contingent on the characteristics of child reactivity. Specifically, compared to low reactive peers, highly reactive children would be affected by maternal personality traits both for worse and for better.

## Materials and Methods

### Participants

The initial sample included 72 families from large communities in Beijing, China, through online recruitment. The selection criteria were: (a) the child was the first-born in the family; (b) the child was born full-term (i.e., at least 37 weeks of pregnancy), (c) the child had no physical or mental disability, and (d) the child was between 12 and 24 months of age. Parental written consent was obtained for all participants. Two families were removed from analysis, due to missingness. Thus, the final sample included 70 children (38 boys and 32 girls), ranging in age from 14 to 22 months (*M* = 17.6 months, *SD* = 3.73).

### Measures

#### Demographic Variables

Demographic characteristics included children’s gender and age, mothers’ education and families’ monthly income. maternal education was coded as 1 for high-school education or lower, 2 for college or professional school degree and 3 for graduate education or higher. The monthly income was coded as 1 (<3000 CNY), 2 (3000–6000 CNY), 3 (6000–10000 CNY) and 4 (>10000 CNY). In the sample, 13.9% of the mothers had a high school or lower education, 61.1% had a college or professional school education, and 25.0% had a graduate education or higher. Monthly family income ranged from: 7.1% of the families earned 3000 CNY or less; 24.3% earned 3000–6000 CNY; 40.0% earned 6000–10000 CNY; and 28.6% earned 10000 CNY or more.

#### Maternal Personality

Maternal personality was measured using the Neuroticism Extraversion Openness Five-Factor Inventory (NEO-FFI), Form S, adapted from Costa and McCrae (1992). This 60-item inventory measures five global domains of personality: Neuroticism, Extraversion, Openness, Agreeableness, and Conscientiousness, each including 12 items. Each participant was requested to rate how well each item described herself, using a 5-point scale, ranging from 1 (strongly disagree) to 5 (strongly agree). Extraversion subscale measures the extent to which the person actively engages in the world or social experiences [e.g., (1) I’d like to have many friends. (2) I like to chat with others]. Agreeableness subscale describes a general willingness to accommodate others. Agreeable people are empathic, altruistic, helpful and trusting [e.g., (1) I try to be polite to everyone I meet. (2) I believe that human nature is kind]. Conscientiousness subscale depicts a concentrated, reliable, high-achieving orientation at work with high involvement and perseverance [e.g., (1) I will try my best to complete all the tasks assigned to me. (2) I have some clear goals]. Neuroticism subscale describes the extent to which the person regards the world as distressing or threatening [e.g., (1) Sometimes I feel angry and full of resentment. (2) I often feel helpless]. Openness to experience is an assessment of the novelty-seeking and the tolerance of unconventionality [e.g., (1) I’d like to raise new hobbies. (2) I am curious about many things]. In the current study, the Cronbach’s alphas of each scale ranged from 0.71 to 0.81.

#### Child Temperamental Reactivity

Child temperamental reactivity was assessed using the Chinese version of the Toddler Temperament Questionnaire (TTQ-CR), established by Carey and McDevitt (1978). The 95-item Chinese version was revised by Zhang et al. (2000). As described above, reactivity denotes reactive intensity and threshold (Rothbart and Bates, 2006). The reaction intensity describes the energy level of response [e.g., (1) My child reacts strongly to failure (such as crying or stamping). (2) My child will cry and scream when encountering difficulties] and the threshold of responsiveness measures the intensity level of stimulation needed to evoke an infant’s response [e.g., (1) My child will immediately ask to change the clothes when they get wet. (2) My child doesn’t pay attention to whether the taste of food is different] Each subscale includes 10 items and each item refers to a particular behavior or characteristic. The scores of child reactivity were calculated as the average of the z-scores of reactive intensity and threshold (Curtindale et al., 2007). Mothers rated children’s daily performance, using a six-point scale from almost never to almost always, with higher scores indicating greater reactivity. Commonly speaking, highly reactive children tend to detect weak stimulation and experience arousal of high intensity. The children with lower scores are insensitive to stimulation. The Cronbach’s alphas were 0.68 for intensity and 0.71 for threshold.

#### Externalizing Behavioral Problems

Using Chinese Version of Infant-Toddler Social and Emotional Assessment (ITSEA-CR) (Jianduan et al., 2009), each mother rated her child’s externalizing behavioral problems on a 3-point scale from 0 (strongly disagree) to 2 (strongly agree). This scale is commonly used to measure social and emotional development of children aged 12–36 months. The externalizing problems were indicated by impulsivity, aggression, and peer aggression. Because the participating children in this study were the first-borns in the family, and at this age, they have limited interactions as with peers, the scores of peer aggressive behaviors were removed from further analysis. Impulsivity [e.g., (1) Crying when he (she) is not satisfied. (2) Too excited to control himself (herself) when my child is playing] and aggression [e.g., (1) Beat or bite parents. (2) Disobedient. For example, he/she is determined to reject when you ask your child to do something] were taken as the two indicators of externalizing behavioral problems in the present study. The Cronbach’s alphas were 0.70 for impulsivity and 0.73 for aggression.

#### Procedure

This study was carried out in accordance with the recommendations of the Research Ethics Committee of Capital Normal University. Written informed consent was obtained from all participants and from the parents/legal guardians of all participants in accordance with the Declaration of Helsinki. The protocol was approved by the Research Ethics Committee of Capital Normal University. The recruit information was posted online and from the families who signed up from this study, we selected 72 families using the criteria mentioned above in the “Participants” section. After making a telephone appointment, two trained research assistants collected the data at each participant’s home. Mothers were asked to complete the NEO-FFI, TTQ-CR and ITSEA-CR, and reported her demographic background information.

#### Analysis

First, we applied Harman’s single-factor test to check method variance (Aulakh and Gencturk, 2000), as all the variables obtained from the mothers’ reports had a potential risk of common method bias. If common method variance indicated a problem, a single factor explaining most of the covariance in the independent and dependent variables would be found in factor analysis. As described in **Table 1**, the result of factor analysis suggested that there were three factors, each with an eigenvalue greater than 1. These results indicated that common method bias was not substantial.

**Table 1:** Results of factor analysis for method variance test.

Factors	Eigenvalue	% of Variance	Cumulative %
1	2.55	28.29	28.29
2	1.81	20.12	48.41
3	1.32	14.62	63.03


Second, descriptive statistics and correlations were presented in **Table 2**. Then, hierarchical multiple regressions were conducted to examine the main effects and interactive effects of maternal personality and child reactivity on children’s impulsivity and aggression, after the predictors were standardized.

**Table 2:** Descriptive statistics and correlations among maternal personality and child behavioral outcome variables.

	1	2	3	4	5	6	7	8
1. Neuroticism	1.00							
2. Extraversion	–0.56^∗∗^	1.00						
3. Openness	–0.24^∗^	0.11	1.00					
4. Agreeableness	–0.29^∗^	0.27^∗^	0.01	1.00				
5. Conscientiousness	–0.40^∗∗^	0.42^∗∗^	–0.10	0.33^∗∗^	1.00			
6. Reactivity	0.07	–0.06	–0.19	–0.01	0.13	1.00		
7. Impulsivity	0.01	0.06	–0.11	–0.36^∗∗^	–0.13	0.09	1.00	
8. Aggression	0.10	–0.09	–0.14	–0.33^∗∗^	–0.15	0.24^∗^	0.66^∗∗^	1.00
M	2.53	3.47	3.08	3.67	3.85	0.00	0.73	0.51
SD	0.65	0.51	0.47	0.35	0.47	0.72	0.50	0.37


*^∗^*p* < 0.05, ^∗∗^*p* < 0.01.*

Finally, the Regions of Significance analysis (RoS) was conducted to evaluate the extent to which the data fits the differential susceptibility model or the diathesis-stress model. This method functions to differentiate the two models in the following steps (Roisman et al., 2012). First, Regions of Significance on X (e.g., family environment; RoS on X) was tested to demonstrate that Y (e.g., children’s development) and Z (children’s reactivity) are correlated at the high and low ends of the distribution of X bounded by a conventional range of interest, that is, ± 2 *SD* from the mean of X. Second, this method yields two indices that are invariant to sample size: the proportion of interaction (PoI) index and the proportion affected (PA) index, to quantify the effects. The value of PoI between 0.40 and 0.60 and PA equal to or greater than 16% indicates an interaction effect consistent with the differential susceptibility model. Finally, because differential susceptibility effects might be an artifact of imposing a linear model on a non-linear model (Roisman et al., 2012), we tested whether the non-linear effect was present using an additional model including X^2^ and Z^∗^X^2^. This analysis of RoS was employed to test the interaction following the instructions available at: *http://www.yourpersonality.net/interaction/*.

## Results

### Preliminary Analysis

Descriptive statistics and bivariate correlations among all variables are shown in **Table 2**. Maternal agreeableness was negatively related to child impulsivity and aggression, while the correlations between other traits of maternal personality and child impulsivity and aggression were not significant. Additionally, child reactivity was positively correlated with child aggression.

### Hierarchical Multiple Regressions

Hierarchical multiple regressions were conducted with child impulsivity and aggression as dependent variables, and maternal personality traits and child reactivity as predictors. Child gender (boy = 0, girl = 1), age, mothers’ education, and family income were entered in Step 1 as control variables. The five traits of maternal personality and child reactivity were entered in Step 2. Because a three-way interaction involving the five traits of maternal personality, child reactivity, and child gender was non-significant, only two-way interaction terms of maternal personality and child reactivity were entered in Step 3. The results of collinearity diagnostics showed that the tolerances < 0.20, VIFs < 5, suggesting that it was not a problem (Fox and Monette, 1992).

As shown in **Table 3**, the regression results revealed that maternal agreeableness was negatively associated with child impulsivity (*b* = -0.14, 95% CI = [-0.26, -0.02], *p* < 0.05). However, maternal neuroticism, extraversion, openness, and conscientiousness were not significantly related to child impulsivity and aggression. The interaction terms of Neuroticism × Child reactivity and Conscientiousness × Child reactivity were statistically significant [*b* = -0.20, 95% *CI* = (-0.40, -0.01), *p* < 0.05; *b* = -0.19, 95% *CI* = (-0.32, -0.06), *p* < 0.05, respectively].

**Table 3:** Hierarchical multiple regressions for children’s externalizing behaviors.

Predictor variable	Impulsivity					Aggression				
	*B* [95% CI]	*SE*	β	*R^2^*	*ΔR^2^*	*B*	*SE*	β	*R^2^*	*ΔR^2^*
**Step 1**										
Child gender	–0.28^∗^ [–0.50, -0.06]	0.11	–0.28	0.21	0.21^∗^	–0.15 [–0.32, 0.03]	0.09	–0.20	0.11	0.11
Child age	–0.01 [–0.04, 0.04]	0.02	–0.07			0.01 [–0.02, 0.03]	0.01	0.06		
Mother education	–0.04 [–0.24, 0.16]	0.10	–0.06			–0.02 [–0.18, 0.14]	0.08	–0.03		
Family income	–0.18^∗^ [–0.33, -0.02]	0.08	–0.32			–0.10 [–0.22, 0.02]	0.06	–0.24		
**Step 2**										
Reactivity	0.06 [–0.06, 0.17]	0.06	0.12	0.33	0.11	0.09^+^ [0.00, 1.76]	0.05	0.23	0.25	0.13
Neuroticism	–0.05 [–0.20, 0.10]	0.07	–0.10			–0.02 [–0.13,0.10]	0.06	–0.05		
Extraversion	0.10 [–0.04, 0.24]	0.07	0.20			0.02 [–0.09, 0.13]	0.05	0.05		
Openness	–0.04 [–0.16, 0.08]	0.06	–0.09			–0.04 [–0.13, 0.06]	0.05	–0.10		
Agreeableness	–0.14^∗^ [–0.26, -0.02]	0.06	–0.28			–0.10^+^ [–0.19, 0.00]	0.05	–0.26		
Conscientiousness	–0.08 [–0.21, 0.05]	0.07	–0.17			–0.05 [–0.16, 0.05]	0.05	–0.14		
**Step 3**										
Neuroticism × Reactivity	–0.20^∗^ [–0.40, -0.01]	0.10	–0.35	0.44	0.11^+^	–0.07 [–0.23, 0.10]	0.08	–0.15	0.31	0.06
Extraversion × Reactivity	0.04 [–0.12, 0.20]	0.08	0.07			0.02 [–0.12, 0.15]	0.07	0.04		
Openness × Reactivity	–0.06 [–0.20, 0.09]	0.07	–0.11			–0.02 [–0.14, 0.10]	0.06	–0.05		
Agreeableness × Reactivity	–0.12 [–0.24, 0.00]	0.06	–0.25			–0.10 [–0.20, 0.01]	0.05	–0.27		
Conscientiousness × Reactivity	–0.19^∗^ [–0.32, -0.06]	0.07	–0.40			–0.07 [–0.17, 0.04]	0.05	–0.19		


*^+^*p* < 0.10, ^∗^*p* < 0.05, ^∗∗^*p* < 0.01. The reported values are from the first time each variable entered the equation.*

### Regions of Significance Analysis

**FIGURE 3:**
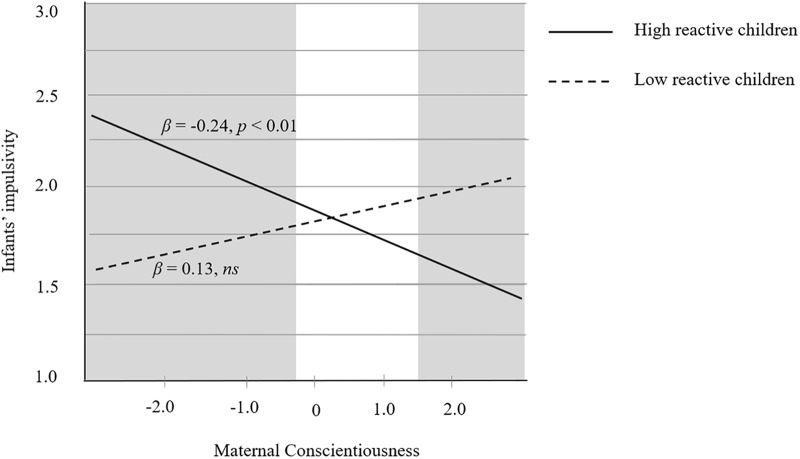
Regression lines for the relationship between maternal conscientiousness and child impulsivity regarding children showing high temperamental reactivity (solid line) and children with low temperamental reactivity (dotted line). Gray shaded areas represent regions of significance (RoS) where the two relationships differ significantly.

The Region of Significance analysis was conducted to test whether the interactive effects fit the differential susceptibility model or diathesis-stress model. In terms of the interaction of Conscientiousness × Child reactivity (see **Table 4** and **Figure 3**), the simple slope analysis showed that maternal conscientiousness negatively predicted infant impulsivity among highly reactive infants (β = -0.24, *p* < 0.05), but not among low reactive infants (β = -0.13, *p* > 0.05). RoS analysis showed that the value of PoI = 0.37 and PA = 0.40. According to Roisman et al. (2012), a differential susceptibility case would have a value between 0.40 and 0.60 for PoI, or PA values equal to or greater than 0.16. In addition, RoS of maternal conscientiousness was [-0.37, 1.46] indicating that the highly reactive infants showed less impulsivity when (1) maternal conscientiousness was above 1.46 (i.e., at high maternal conscientiousness) and (2) child impulsivity was below -0.37 (i.e., at low maternal conscientiousness) than the low reactive infants. Moreover, neither X^2^, nor Z^∗^X^2^, nor a combination of both non-linear terms together was statistically significant, suggesting that there was no non-linear relation between the variables. In brief, all these statistical indices of the Conscientiousness × Child reactivity provided support for the differential susceptibility model.

**Table 4:** ROS indices for statistically significant maternal personality × child reactivity interactions.

Interaction	RoS X		PoI	PA	Crossover	X^2^ or ZX^2^
	Lower bound	Upper bound				
Neuroticism × Reactivity	–0.45	7.78	0.38	0.41	0.24	*Ns*
Conscientiousness × Reactivity	–0.37	1.46	0.37	0.40	0.26	*Ns*


*RoS X, the regions of significance with respect to maternal personality; PoI (Proportion of Interaction), the proportion of the interaction that fell above the cross-over point for the regressions; PA (Proportion Affect), the proportion of participants who had early sensitivity scores that fell above the crossover point; X^2^ or ZX^2^, were used to test the non-linear relations among the variables; *Ns*, not significant.*

**FIGURE 4:**
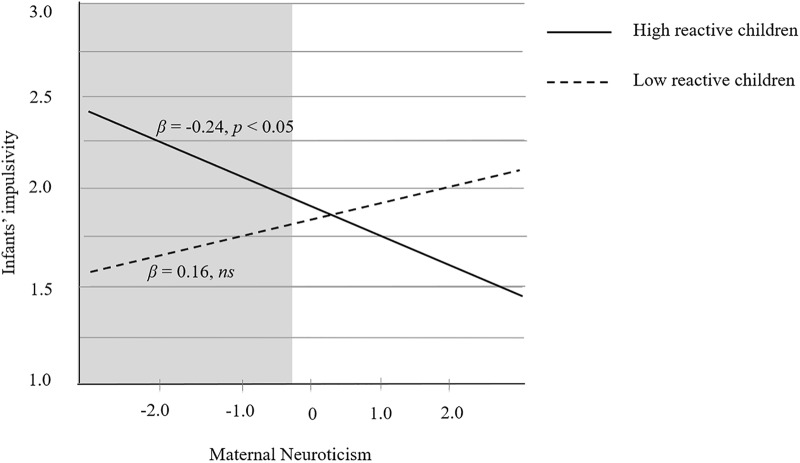
Regression lines for the relationship between maternal Neuroticism and child impulsivity regarding children showing high temperamental reactivity (solid line) and children with low temperamental reactivity (dotted line). Gray shaded areas represent RoS where the two relationships differ significantly.

In terms of the interaction of Neuroticism × Child reactivity (see **Table 4** and **Figure 4**), the simple slope analysis showed that maternal neuroticism negatively predicted infant impulsivity among highly reactive infants (β = -0.24, *p* < 0.05), but not among the low reactive infants (β = -0.16, *p* > 0.05). RoS analysis showed that the value of PoI = 0.38 and PA = 0.41, in support of the differential susceptibility model. The RoS of maternal neuroticism was [-0.45, 7.78] indicating that compared to their low reactive counterparts, highly reactive children showed more impulsivity when the value of maternal neuroticism was below -0.45 (i.e., at low maternal neuroticism). The upper bound fell outside the recommended range in validating the diathesis-stress hypothesis. Therefore, the results failed to support either the diathesis-stress or the differential susceptibility model.

## Discussion

The main purposes of the current study were to explore the effects of maternal personality on child externalizing problems and the moderating role of child temperamental reactivity in the associations between maternal personality and child externalizing behavioral problems, in a sample of Chinese children aged 12–24 months. The findings extended the existing literature concerning the associations between maternal personality and child behavioral problems, supporting the differential susceptibility model. Specifically, it is suggested that maternal personality appears to be a significant socialization factor in child development, and its contributions to children’s externalizing behavioral problems might depend on the characteristics of child temperamental reactivity.

### Relations Between Maternal Personality and Child Externalizing Behavioral Problems

The findings indicated that maternal agreeableness was negatively associated with child impulsivity irrespective of the level of child temperamental reactivity. Children with highly agreeable mothers, who are altruistic, sympathetic, kind and willing to help, tend to display less impulsivity. We speculated that, from the perspective of social learning theory, highly agreeable mothers might present themselves as a role model to their children on how to cooperate with and positively respond to others. The social skills thus learned would in turn help children, regardless of their reactivity, display low impulsivity in interpersonal interactions (Cipra, 2018).

We also found that maternal neuroticism, extraversion, openness, and conscientiousness were not directly related to child externalizing behavioral problems in the present study. This is in contrast with previous findings. For example, Prinzie et al. (2004, 2005) found that maternal extraversion and conscientiousness were negatively associated with their children’s behavioral problems while paternal openness to new experiences was positively related to children’s antisocial behavior. There are two possible explanations for the inconsistent results. First, the impact of maternal personality on child development may vary by child age. Relative to school-aged children or adolescents, younger children tend to show less variations in their behavioral adjustment led by the influence of socialization. Second, the associations between maternal personality and children’s externalizing behavioral problems might be moderated by other child characteristics, such as temperament. Therefore, we could not draw the conclusion that maternal neuroticism, extraversion, openness, and conscientiousness did not predict child externalizing behavioral problems because of their non-significant main effects. It is possible that these traits of maternal personality might have different effects on susceptible children and their main effects might be embodied in the interactions between maternal personality and child characteristics. For instance, our findings showed that maternal conscientiousness and neuroticism were not directly associated with child impulsivity. However, maternal conscientiousness and neuroticism jointly affected highly reactive children, but not low reactive children, indicating the role of child reactivity in moderating the joint relations.

### The Moderating Role of Child Reactivity

The moderating effects of child temperamental reactivity were examined in the current study. Consistent with previous research, findings on the interaction patterns between conscientiousness and child reactivity on externalizing problems were in support of the differential susceptibility model (Ramchandani et al., 2010; Gueronsela et al., 2016; Xing et al., 2016). For highly reactive children, having a conscientious mother negatively predicted their behavioral impulsivity but this prediction was not revealing in low reactive children. Why only highly reactive children were affected by maternal conscientiousness? Perhaps, due to the underlying reactivity of their neural systems, highly reactive children are relatively more sensitive to external stimulations than others (Ramchandani et al., 2010). Mothers high on conscientiousness tend to be orderly, responsible and dependable. They are likely to have high standards in parenting and to feel obliged to respond and support their children under most circumstances, which includes regulating children’s impulsive behaviors in response to emotional arousal (Slagt et al., 2015). On the contrary, low conscientious mothers may be less attentive and supportive and more ambiguous in parenting (Clark et al., 2000), which in turn results in behavioral and emotional malfunctioning in highly reactive children. It is also possible that in the reciprocal relationships between maternal parenting behaviors and child behaviors. That is, children’s impulsivity and other undercontrolling behaviors would have an impact on how mothers evaluate their parental strategies and interact with them in daily activities (Belsky, 1984). From this perspective, one might expect that the impulsivity exhibited in highly reactive children would provoke the unconscientious mothers to be more frustrated and thus less involved in parenting, which in turn might result in more dysregulated behaviors in children.

In addition, there was also an interaction between maternal neuroticism and child reactivity on impulsivity, although not all statistical indices supported the differential susceptibility model. Further analysis showed a significantly negative association between maternal neuroticism and impulsivity in highly reactive children. This result is consistent with previous findings. For instance, some studies found that maternal neuroticism was positively related to social withdrawal (Ellenbogen and Hodgins, 2004) and inhibition (Belsky and Barends, 2002) in children. Moreover, lower maternal emotional stability was related with higher children’s social wariness (Degnan et al., 2008). Considering these findings, it is understandable that maternal neuroticism was negatively related with behavioral impulsivity in highly reactive children because of their sensitive neural systems.

Finally, maternal personality did not significantly predict child aggression in the current study, which might be related to the age differences in the prevalence of aggression. Empirical research suggested that although the majority of children first reached the onset of aggressive behavior at around 17 months of age, it occurs significantly more often at 24- to 36-months (e.g., Hay et al., 2000; Alink et al., 2010). Therefore, unlike in young children as those in the present study, it is possible that the effects of maternal personality on child aggression might be salient among older children. Given that, more studies with a wider age range are needed to replicate and extend the present findings.

### Limitations and Directions for Future Study

The current study was the first to examine the joint effects of maternal personality and child temperamental reactivity on child externalizing behavioral problems. Several strengths in this study are noticeable. For example, the subjects were toddlers and their mothers, which extended previous studies in the field that were mostly conducted in early childhood. Moreover, the RoS analysis allowed us to examine the interactive effects between maternal personality and child temperamental reactivity more precisely. Nevertheless, there are also some limitations that should be acknowledged.

First, the data of this study were collected using mothers reports, which might partly reflect the reporter bias and subjective judgments and increase the risk of common method variance. Moreover, the sample size was relatively small which might lead to low statistical power. Additionally, data in this study were cross-sectional, which does not allow us to conclusively identify the direction of the association between maternal personality and children’s externalizing problems and the trajectory of the effects of maternal personality on children’s behavioral problems in different developmental stages. Thus, to achieve a more comprehensive understanding of the issues, it will be important in future research to use a longitudinal design with a larger sample size.

Second, the results revealed interactions only between maternal conscientiousness, neuroticism and child reactivity. There might be interplay effects between maternal personality traits and other susceptible characteristics in children, such as premature birth (Gueronsela et al., 2016) and negative emotionality (Morgan et al., 2012). Therefore, future research should explore the moderating roles of other susceptible factors in children (i.e., negative emotionality) in the associations between maternal personality and child development.

Finally, although there was clear evidence on the interaction between maternal personality and child reactivity on externalizing behavioral problems, the mechanisms concerning the moderation effects were not explored. There are studies suggesting that parental personality may shape parenting behaviors which may contribute to the quality of parent-child interactions and children’s developmental outcomes (Belsky, 1984; Clark et al., 2000; Coplan et al., 2009). Hence, future research is needed to examine the mechanisms by which parental personality contributes to children’s externalizing behavioral problems.

## Author Contributions

ZW, SX, and XL conceived and designed the study. XG and YM performed the collection and analysis of data. SX, XG, XL, and YM completed and modified the manuscript.

## Conflict of Interest Statement

The authors declare that the research was conducted in the absence of any commercial or financial relationships that could be construed as a potential conflict of interest.

## References

[B1] Achtergarde S. Postert C. Wessing I. Romer G. Muller J. M. (2015). Parenting and child mental health influences of parent personality, child temperament, and their interaction. *Fam. J.* 23 167–179. 10.1177/1066480714564316 23218244

[B2] Alink L. R. Mesman J. Van Z. J. Stolk M. N. Juffer F. Koot HM (2010). The early childhood aggression curve: development of physical aggression in 10- to 50-month-old children. *Child Dev.* 77 954–966. 10.1111/j.1467-8624.2006.00912.x 16942499

[B3] Aulakh P. S. Gencturk E. F. (2000). International principal–agent relationships: control, governance and performance. *Ind. Mark. Manag.* 29 521–538. 10.1016/S0019-8501(00)00126-7

[B4] Bakermans-Kranenburg M. J. Ijzendoorn M. H. V. (2007). Research review: genetic vulnerability or differential susceptibility in child development: the case of attachment. *J. Child Psychol. Psychiatry* 48 1160–1173. 10.1111/j.1469-7610.2007.01801.x 18093021

[B5] Belsky J. (1984). The determinants of parenting: a process model. *Child Dev.* 55 83–96. 10.2307/1129836 6705636

[B6] Belsky J. (1997). Variation in susceptibility to environmental influence: an evolutionary argument. *Psychol. Inq.* 8 182–186. 10.1207/s15327965pli0803_3

[B7] Belsky J. Barends N. (2002). “Personality and parenting,” in *Handbook of Parenting: Being and Becoming a Parent*, ed. Bornstein M. H. (Mahwah, NJ: Lawrence Erlbaum Associates Publishers), 415–438.

[B8] Belsky J. Pluess M. (2009). Beyond diathesis stress: differential susceptibility to environmental influences. *Psychol. Bull.* 135 885–908. 10.1037/a0017376 19883141

[B9] Belsky J. Pluess M. (2013). Beyond risk, resilience, and dysregulation: phenotypic plasticity and human development. *Dev. Psychopathol.* 25 1243–1261. 10.1017/S095457941300059X 24342838

[B10] Bulotskyshearer R. J. Fantuzzo J. W. (2011). Preschool behavior problems in classroom learning situations and literacy outcomes in kindergarten and first grade. *Early Child. Res. Q.* 26 61–73. 10.1016/j.ecresq.2010.04.004

[B11] Calkins S. D. Gill K. L. Williford A. P. (1999). Externalizing problems in two-year-olds: implications for patterns of social behavior and peers’ responses to aggression. *Early Edu. Dev.* 10 267–288. 10.1207/s15566935eed1003_3

[B12] Carey W. B. McDevitt S. C. (1978). Revision of the infant temperament questionnaire. *Pediatrics* 61 735–739. 662513

[B13] Cipra A. (2018). Differential susceptibility and kindergarten peer status. *Child Indicators Res.* 1–21. 10.1007/s12187-018-9545-4

[B14] Clark L. A. Kochanska G. Ready R. E. (2000). Mothers’ personality and its interaction with child temperament as predictors of parenting behavior. *J. Pers. Soc. Psychol.* 79 274–285. 10.1037/0022-3514.79.2.274 10948980

[B15] Coie J. D. Dodge K. A. (1998). “Aggression and antisocial behavior,” in *Handbook of Child Psychology* Vol. 3 eds Damon W. Eisenberg N. (New York, NY: Wiley), 779–862.

[B16] Coplan R. J. Reichel M. Rowan K. (2009). Exploring the associations between maternal personality, child temperament, and parenting: a focus on emotions. *Pers. Individ. Differ.* 46 241–246. 10.1016/j.paid.2008.10.011

[B17] Cormier E. (2008). Attention deficit/hyperactivity disorder: a review and update. *J. Pediatr. Nurs.* 23 345–357. 10.1016/j.pedn.2008.01.003 18804015

[B18] Costa P. T. McCrae R. R. (1992). Four ways five factors are basic. *Pers. Individ. Differ.* 13 653–665. 10.1016/0191-8869(92)90236-I

[B19] Curtindale L. M. Laurierose C. Bennettmurphy L. Hull S. (2007). Sensory modality, temperament, and the development of sustained attention: a vigilance study in children and adults. *Dev. Psychol.* 43 576–589. 10.1037/0012-1649.43.3.576 17484572

[B20] Degnan K. A. Henderson H. A. Fox N. A. Rubin K. H. (2008). Predicting social wariness in middle childhood: the moderating roles of child care history, maternal personality and maternal behavior. *Soc. Dev.* 17 471–487. 10.1111/j.1467-9507.2007.00437.x 20463856 PMC2867489

[B21] Den Berg H. V. Bus A. G. (2014). Beneficial effects of bookstart in temperamentally highly reactive infants. *Learn. Individ. Differ.* 36 69–75. 10.1016/j.lindif.2014.10.008

[B22] Ellenbogen M. A. Hodgins S. (2004). The impact of high neuroticism in parents on children’s psychosocial functioning in a population at high risk for major affective disorder: a family-environmental pathway of intergenerational risk. *Dev. Psychopathol.* 16 113–136. 10.1017/S0954579404044438 15115067

[B23] Ellis B. J. Boyce W. T. Belsky J. Bakermanskranenburg M. J. Van Ijzendoorn M. H. (2011). Differential susceptibility to the environment: an evolutionary–neurodevelopmental theory. *Dev. Psychopathol.* 23 7–28. 10.1017/S0954579410000611 21262036

[B24] Fox J. Monette G. (1992). Generalized collinearity diagnostics. *J. Am. Stat. Assoc.* 87 178–183. 10.1080/01621459.1992.10475190

[B25] Gueronsela N. Atzabaporia N. Meiri G. Marks K. (2016). Temperamental susceptibility to parenting among preterm and full-term infants in early cognitive development. *Infancy* 21 312–331. 10.1111/infa.12120

[B26] Hay D. F. Castle J. Davies L. (2000). Toddlers’ use of force against familiar peers: a precursor of serious aggression? *Child Dev.* 71 457–467. 10834477 10.1111/1467-8624.00157

[B27] Jianduan Z. Huishan W. Shuhua S. Xiaonan H. Guoyan L. Guangli L. (2009). Reliability and validity of standardized Chinese version of urban infant-toddler social and emotional assessment. *Early. Hum. Dev.* 85 331–336. 10.1016/j.earlhumdev.2008.12.012 19181462

[B28] Keenan K. Wakschlag L. S. (2000). More than the terrible twos: the nature and severity of behavior problems in clinic-referred preschool children. *J. Abnorm. Child Psychol.* 28 33–46. 10.1023/A:1005118000977 10772348

[B29] Koutra K. Roumeliotaki T. Kyriklaki A. Kampouri M. Sarri K. Vassilaki M. (2017). Maternal depression and personality traits in association with child neuropsychological and behavioral development in preschool years: mother-child cohort (Rhea Study) in Crete, Greece. *J. Affect. Disord.* 217:89. 10.1016/j.jad.2017.04.002 28395209

[B30] Morgan J. K. Shaw D. S. Olino T. M. (2012). Differential susceptibility effects: the interaction of negative emotionality and sibling relationship quality on childhood internalizing problems and social skills. *J. Abnorm. Child Psychol.* 40 885–899. 10.1007/s10802-012-9618-7 22366882 PMC3411103

[B31] Oliver P. H. Guerin D. W. Coffman J. K. (2009). Big five parental personality traits, parenting behaviors, and adolescent behavior problems: a mediation model. *Pers. Individ. Differ.* 47 631–636. 10.1016/j.paid.2009.05.026

[B32] Prinzie P. Onghena P. Hellinckx W. Grietens H. Ghesquiere P. Colpin H. (2004). Parent and child personality characteristics as predictors of negative discipline and externalizing problem behaviour in children. *Eur. J. Pers.* 18 73–102. 10.1002/per.501

[B33] Prinzie P. Onghena P. Hellinckx W. Grietens H. Ghesquiere P. Colpin H. (2005). Direct and indirect relationships between parental personality and externalising behaviour: the role of negative parenting. *Psychol. Belgica* 45 123–145. 10.5334/pb-45-2-123

[B34] Ramchandani P. G. Jzendoorn M. V. I. Bakermanskranenburg M. J. (2010). Differential susceptibility to fathers’ care and involvement: the moderating effect of infant reactivity. *Fam. Sci.* 1 93–101. 10.1080/19424621003599835 22073318 PMC3208580

[B35] Roisman G. I. Newman D. A. Fraley R. C. Haltigan J. D. Groh A. M. Haydon K. C. (2012). Distinguishing differential susceptibility from diathesis–stress: recommendations for evaluating interaction effects. *Dev. Psychopathol.* 24 389–409. 10.1017/S0954579412000065 22559121

[B36] Rothbart M. K. Bates J. E. (2006). “Temperament,”. in *Handbook of child psychology: Social, Emotional, and Personality Development*, eds Damon W. Lerner R. M. Eisenberg N. (New York, NY: Wiley), 99–166.

[B37] Slagt M. Dubas J. S. Denissen J. J. A. Dekovic M. Van Aken M. A. G. (2015). Personality traits as potential susceptibility markers: differential susceptibility to support among parents. *J. Pers.* 83 155–166. 10.1111/jopy.12091 24471708

[B38] Super C. M. Harkness S. (1986). The developmental niche: a conceptualization at the interface of child and culture. *Int. J. Behav. Dev.* 9 545–569. 10.1177/016502548600900409

[B39] Thartori E. Zuffianò A. Pastorelli C. Di Giunta L. Lunetti C. Lansford J. E. (2018). The interactive effects of maternal personality and adolescent temperament on externalizing behavior problem trajectories from age 12 to 14. *Pers. Individ. Differ.* 134 301–307. 10.1016/j.paid.2018.06.021

[B40] Van Hulle C. A. Rodgers J. L. Donofrio B. M. Waldman I. D. Lahey B. B. (2007). Sex differences in the causes of self-reported adolescent delinquency. *J. Abnorm. Psychol.* 116 236–248. 10.1037/0021-843X.116.2.236 17516757

[B41] Velderman M. K. BakermansKranenburg M. J. Juffer F. van IJzendoorn M. H. (2006). Effects of attachment-based interventions on maternal sensitivity and infant attachment: differential susceptibility of highly reactive infants. *J. Fam. Psychol.* 20 266–274. 10.1037/0893-3200.20.2.266 16756402

[B42] Xing S. Zhou Q. Archer M. Yue J. Wang Z. (2016). Infant temperamental reactivity, maternal and grandparental sensitivity: differential susceptibility for behavior problems in China. *Early. Hum. Dev.* 101 99–105. 10.1016/j.earlhumdev.2016.08.014 27614331

[B43] Zhang J. S. Xu J. D. Shen L. X. (2000). The assessment of Carey’s five temperament questionnaires in one-month-old to twelve-year old children. *Chin. Ment. Health J.* 14 153–156.

